# Pore-Scale Modeling of Liquid Water Transport in Compressed Gas Diffusion Layer of Proton Exchange Membrane Fuel Cells Considering Fiber Anisotropy

**DOI:** 10.3390/membranes13060559

**Published:** 2023-05-29

**Authors:** Hao Wang, Guogang Yang, Shian Li, Qiuwan Shen, Yue Li, Renjie Wang

**Affiliations:** 1 Laboratory of Transport Pollution Control and Monitoring Technology, Beijing 100084, China; whdlmu@dlmu.edu.cn (H.W.); lishian@dlmu.edu.cn (S.L.);; 2 Marine Engineering College, Dalian Maritime University, Dalian 116026, China; shenqiuwan@dlmu.edu.cn

**Keywords:** gas diffusion layer, water management, compression, fiber anisotropy, lattice Boltzmann method

## Abstract

Water management of the gas diffusion layer (GDL) is crucial to the performance of proton exchange membrane fuel cells (PEMFCs). Appropriate water management ensures efficient transport of reactive gases and maintains wetting of the proton exchange membrane to enhance proton conduction. In this paper, a two-dimensional pseudo-potential multiphase lattice Boltzmann model is developed to study liquid water transport within the GDL. Liquid water transport from the GDL to the gas channel is the focus, and the effect of fiber anisotropy and compression on water management is evaluated. The results show that the fiber distribution approximately perpendicular to the rib reduces liquid water saturation within the GDL. Compression significantly changes the microstructure of the GDL under the ribs, which facilitates the formation of liquid water transport pathways under the gas channel, and the increase in the compression ratio leads to a decrease in liquid water saturation. The performed microstructure analysis and the pore-scale two-phase behavior simulation study comprise a promising technique for optimizing liquid water transport within the GDL.

## 1. Introduction

A proton exchange membrane fuel cell (PEMFC) is an energy device that converts the chemical energy in hydrogen and oxygen directly into electrical energy and has attracted a lot of attention for its high efficiency, zero emissions, and low noise [1]. At the PEMFC cathode, oxygen flows from the gas flow path through the gas diffusion layer and reacts with protons in the catalytic layer to produce water. Improper water management can cause problems such as membrane dehydration and flooding, which can seriously affect the performance and lifetime of the fuel cell [2]. The main role of the proton exchange membrane in a fuel cell is to achieve rapid proton conduction and also to block the permeation of hydrogen and oxygen and nitrogen between the cathode and anode. Ideal proton exchange membranes need to have high proton conductivity; low electron conductivity; low gas permeability; and good chemical, electrochemical, and thermal stability [3]. Suitable water management is required to keep the proton exchange membrane humidified to enhance proton conduction and ensure proton exchange membrane performance [4].

The gas diffusion layer (GDL) is an important component of the fuel cell and is usually composed of porous materials with good electrical conductivity and an anisotropic porous microstructure [5,6]. GDLs perform a variety of roles such as gas and water transport, electron conduction, and support for the catalytic layer in fuel cell stacks [7]. The transport of liquid water within the GDL is influenced by its microscopic pore structure, and experimental studies have demonstrated the significant effect of the structural parameters and material characteristics of the GDL on the liquid water transport [8]. Wettability [9], the micro-porous layer [10], and deformation [11] are among the factors that scholars have focused on to influence the dynamics of liquid water. Experimental studies consider realistic GDLs but are too demanding in terms of time and capital costs, so adopting numerical simulation methods, especially pore-scale simulation techniques that can capture the microstructure of GDLs, can more reliably be used to analyze changes in liquid water transport within GDLs due to changes in structural parameters [12,13].

The lattice Boltzmann method is a numerical simulation method that considers particle migration and collisions at the mesoscopic scale and obtains macroscopic quantities through the evolution of the distribution functions of the particles. The complex microstructure and small pore geometry of GDLs make the two-phase flow inside GDLs capillary force driven, and the LBM method can easily introduce intermolecular interactions and capture the interface, so many scholars have adopted LBM to study gas–liquid two-phase transport processes inside GDLs. The liquid water dynamics within the GDL at different rib widths was investigated by Yang et al. [14], and the results showed that PEMFC water management was stronger for the narrower ribs. Similar work was carried out by Jeon [15], which demonstrated that narrower ribs lead to shorter breakthrough times for liquid water into the gas channel. The liquid water removal within the GDL was focused on by Molaeimanesh et al., and the effect of PTFE distribution [16] and wettability gradients [17] was evaluated. Kim et al. [18] and Sepe et al. [19] found that microporous layers can reduce the number of breakthrough sites for liquid water within the GDL and reduce the time for liquid water distribution to reach a steady state. Additionally, LBM has also been applied to study transport parameters such as the permeability [20], diffusivity [21], and thermal conductivity [22] of GDL.

The anisotropy of the fiber changes the fiber arrangement characteristics and the distribution of pore space, affecting the water–gas two-phase transport characteristics. GDLs with different anisotropy differ in their ability to conduct fluids in the through-plane direction and in-plane direction, resulting in different permeability, diffusivity, and water removal performance of the GDL. Equations for the prediction of tortuosity and diffusion coefficients for GDL with different fiber inclination angles were summarized by Espinoza et al. [23]. A GDL reconstruction method considering fiber orientation was proposed by Simaafrookhteh et al. [24] and the permeability of the GDL was evaluated for through-plane and in-plane directions. Gao et al. [25] stochastically reconstructed GDLs with different fiber tilt angles and found that the greater the tilt angle, the greater the permeability of the GDL. Multi-component gas flow within three GDLs with different anisotropy was simulated by Molaeimanesh et al. [26], and it was found that the fiber distribution perpendicular to the rib resulted in a more inhomogeneous current density distribution on the catalyst layer surface. Previous studies on the effect of fiber anisotropy on the mass transfer characteristics of GDLs have mainly focused on the gas flow within the GDL, with very little research on water management. The influence of different fiber orientations on the transport of liquid water within the GDL was investigated by Lee et al. [27]. The results showed that fiber orientation changes the pore distribution and has a significant effect on water transport.

The mechanical stresses introduced by the manufacturing and assembly process can lead to compression of the GDL, resulting in different fiber distribution characteristics below the channels and below the ribs. The effect of compression on the behavior of the two phases within the GDL has been focused on. Kulkarni et al. [28] observed the dynamics of water transport within GDLs with different degrees of compression using neutron radiography and found that compression resulted in a reduction in the efficiency of liquid water removal and an enhancement in water back diffusion. Zenyuk et al. [29] observed the distribution of liquid water within the compressed GDL using X-ray computed tomography, and there was a significant difference in the local liquid water saturation of the GDL in the region below the rib and in the region below the gas channel. The finite volume method was adopted by He et al. [30] to study the crossover behavior of water, and it was found that the compression of the GDL led to a non-uniform distribution of local current density. Kanda et al. [31] found that local stress concentrations at the edges of the ribs affected the current density distribution and water behavior in the GDL. Pore-scale studies on compressed GDLs have focused on transport parameters [32,33,34] and liquid water management [35,36,37,38,39]. The two types of GDLs, namely, Toray TGP-H-060 and Freudenberg H2315, were reconstructed by Zhang et al. [32], and the anisotropic diffusion coefficient, permeability, and electrical and thermal conductivity of the GDLs were evaluated for different compression ratios. The binder and PTFE were considered in the digital model of the compressed GDL developed by Burganos et al. [33], and anisotropic permeability was evaluated. A two-dimensional two-phase LB model was developed by Jeon et al. [35] to study the behavior of liquid water transport within the GDL at different compression ratios. The effects of rib structure and wettability on water transport within the compressed GDL were considered by Lee et al. [36] and Ira et al. [37], respectively. Shojaeefard and Molaeimanesh et al. [38,39] investigated the removal of liquid water from a compressed GDL and showed that low compression ratios enhanced liquid water removal, while high compression ratios reduced the efficiency of liquid water removal.

Research on anisotropic GDLs has not been sufficiently studied, especially the transport mechanisms of liquid water within different anisotropic GDLs. In addition, compression is an inevitable operating condition in practical fuel cell operation, but the behavior of liquid water within the compressed anisotropic GDL has not been reported. In this paper, a two-dimensional pseudopotential two-phase LB model is established to simulate the process of liquid water flowing in the GDL and breaking through into the gas channel. The effects of fiber anisotropy and compression on the dynamics of liquid water in the GDL are considered, the distribution of liquid water is given, and variation of the saturation of liquid water with time is analyzed. This paper provides suggested strategies for the structural design of the GDL, which is helpful to improve the water management of fuel cells.

## 2. Multi-Phase LB Model

For two-phase flow simulation, compared to computational fluid dynamics (CFD) methods, LBM can accurately describe different phases without capturing the phase interface. Currently, the main LB models for simulating multi-phase flows are the color model [40], the pseudo-potential model [41], and the free energy model [42]. In this paper, the pseudo-potential model was used to simulate the two-phase flow within the GDL, which can realize the process of phase separation without additional interface capture functions and describe the interaction between phases in the form of intuitive inter-particle forces, which can better reflect the physical nature of phase separation. A single relaxation time LB model was adopted to study the two-phase flow within the GDL with two sets of distribution functions to describe the water and gas phases, the lattice Boltzmann equation of which can be expressed as [43]

(1) $$f_{i}^{k}(x+c_{i}\Delta t,t+\Delta t)=f_{i}^{k}(x,t)+\frac{\Delta t}{\tau^{k}}\left[f_{i}^{k,eq}(x,t)-f_{i}^{k}(x,t)\right]$$

where $f_{i}^{k}$ denotes the velocity distribution function, and *τ^k^* is the relaxation time of component *k*; here, relaxation time for both water and gas is 1. *f_i_^k,eq^* is the equilibrium distribution function, which can be expressed as follows:

(2) $$f_{i}^{k,eq}=w_{i}\rho^{k}\left[1+\frac{c_{i}\cdot u}{c_{s}{}^{2}}+\frac{1}{2}\frac{{\left(c_{i}\cdot u\right)}^{2}}{c_{s}{}^{4}}-\frac{1}{2}\frac{u^{2}}{c_{s}{}^{2}}\right]$$

where *ρ^k^* is the density and *c_s_* the lattice speed of sound, and *c_s_*^2^ = 1/3. *w_i_* and *c_i_* are the weight factor and particle velocity vector, respectively. The D2Q9 model is adopted in this paper, with 9 possible velocity directions for the particles at each lattice point. They can be given as

(3) $$w_{i}=\{\begin{array}{c} \begin{array}{l} 4/9\;\;\;\;\;i=1\\ 1/9\;\;\;\;\;i=2,3,4,5 \end{array}\\ 1/36\;\;\;i=6,7,8,9 \end{array}$$

(4) $$\begin{array}{ccc} \begin{array}{ccc} \begin{array}{cccc} \begin{array}{cc} {}[c_{1} & c_{2} \end{array} & c_{3} & c_{4} & c_{5} \end{array} & c_{6} & c_{7} \end{array} & c_{8} & c_{9}] \end{array}=\left[\begin{array}{c} \begin{array}{cccc} \begin{array}{cccc} \begin{array}{ccc} 0 & 1 & 0 \end{array} & -1 & 0 & 1 \end{array} & -1 & -1 & 1 \end{array}\\ \begin{array}{cccc} \begin{array}{cccc} \begin{array}{ccc} 0 & 0 & 1 \end{array} & 0 & -1 & 1 \end{array} & 1 & -1 & -1 \end{array} \end{array}\right]$$

The density and velocity (***u****^k^*) of each fluid can be obtained from their respective distribution functions. The equilibrium velocity (***u****^k^*^,*eq*^) in the equilibrium distribution function is redefined in the following way:

(5) $$\rho^{k}=\sum_{i=1}^{9}f_{i}^{k}(x,t)$$

(6) $$\rho^{k}u^{k}=\sum_{i=1}^{9}f_{i}^{k}(x,t)c_{i}$$

(7) $$u^{k,eq}=u^{\prime }+\frac{\tau^{k}F^{k}}{\rho^{k}}$$

where ***u′*** is the combined velocity of the mixture and can be expressed as follows:

(8) $$u^{\prime }=\frac{\sum_{k}\frac{1}{\tau^{k}}\sum_{i=1}^{9}f_{i}^{k}(x,t)c_{i}}{\sum_{k}\frac{1}{\tau^{k}}\rho^{k}}$$

***F****^k^* denotes total external forces, including the fluid–fluid interaction force ($F_{f}^{k}$) and the fluid–solid interaction force ($F_{s}^{k}$). The fluid–fluid interaction force is defined as

(9) $$F_{f}^{k}(x)=-\psi^{k}(\rho^{k}(x))\sum_{x^{\prime }}\sum_{\bar{k}}^{s}G^{k\bar{k}}(x,x^{\prime })\psi^{\bar{k}}(\rho^{\bar{k}}(x))(x^{\prime }-x)$$

where *ψ^k^* is the effective density and can be taken as $\psi^{k}(\rho^{k}(x))=\rho_{0}(1-\exp(-\rho^{k}/\rho_{0}))$. *x′* represents the surrounding nodes centered on position *x*. $G^{k\bar{k}}$ is Green’s function, and when the interaction forces between adjacent and sub-adjacent lattice points are considered, it can be expressed as:

(10) $$G^{k\bar{k}}=\{\begin{array}{c} \begin{array}{l} \;4g\left|x-x^{\prime }\right|=1\\ \;\;\;g\left|x-x^{\prime }\right|=\sqrt{2} \end{array}\\ 0\left|x-x^{\prime }\right|=0 \end{array}$$

Usually, *g* takes a positive value and can be used to adjust the surface tension between different fluids. The fluid–solid interaction force can be expressed as

(11) $$F_{s}^{k}=-\psi^{k}(\rho^{k}(x))\sum_{x^{\prime }}W(x,x^{\prime })s(x^{\prime })(x^{\prime }-x)$$

(12) $$W=\{\begin{array}{c} \begin{array}{l} \;4w\left|x-x^{\prime }\right|=1\\ \;\;\;w\left|x-x^{\prime }\right|=\sqrt{2} \end{array}\\ 0\left|x-x^{\prime }\right|=0 \end{array}$$

where the values of *s*(*x’*) are 0 or 1 for fluid or solid at position *x’*, respectively. *w* adjusts the interaction forces between fluid and solid. More details of the multi-phase LB model adopted in this paper can be found in our previous work [44], and the accuracy has also been validated.

## 3. Two-Dimensional Reconstruction of GDL and Computational Domain

**Figure 1:**
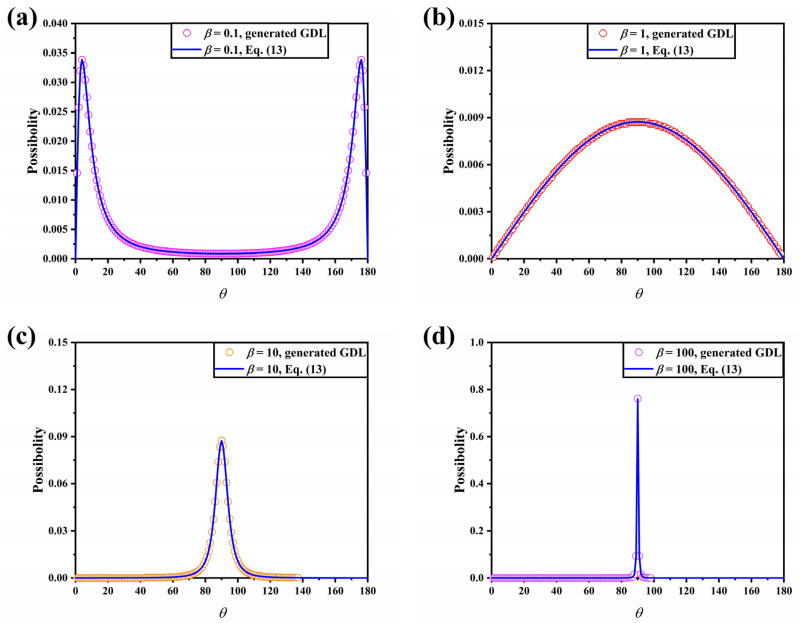
The distribution of the polar angles of the fibers in the four generated 3D GDLs: (**a**) *β* = 0.1; (**b**) *β* = 1; (**c**) *β* = 10; and (**d**) *β* = 100.

To model the two-phase behavior within the GDL, the digital microstructure of the GDL needs to be obtained. There are two general methods of obtaining GDL structures: scanning imaging and stochastic reconstruction. Some scholars have used X-rays, for example, to scan the GDL to obtain a series of consecutive images and then integrate them to obtain the 3D digital structure of the GDL [45]. The stochastic reconstruction method, on the other hand, is based on the statistical information of the GDL and algorithmically reconstructs the structure of the GDL. The stochastic reconstruction method has been the choice of many scholars due to its low cost and ease of implementation and is also employed in this paper. The method of determining fiber orientation from the probability density function of altitude proposed by Schladitz et al. [46] is widely used to generate the GDL:

(13) $$p(\theta )=\frac{1}{4\pi }\frac{\beta \sin\theta }{{\left(1+(\beta^{2}-1)\cos\theta \right)}^{1.5}}$$

where *β* is used to describe the anisotropy of the GDL; the closer *β* tends to zero, the more perpendicular the fibers are to the xy-plane, and the larger *β* is, the more parallel the fibers are to the xy-plane. *θ* is the polar angle. Some assumptions need to be made: the fibers are infinitely long cylinders of equal diameter and are allowed to overlap. In previous work, the anisotropy factor of SGL10BA was reported to be *β* = 100 [26]. Zhang et al. reported an anisotropy factor of *β* = 5 corresponding to Toray TGP-H-060 [32]. Molaeimanesh et al. reconstructed GDLs with *β* = 0.01, 1, and 10,000 to study cathode performance [26]. In this paper, the effect of the anisotropic distribution of carbon fibers on the transport of liquid water was evaluated, and therefore four 3D GDLs of dimensions 2000 × 2000 × 197 μm^3^ with *β* = 0.1, 1, 10, and 100 were generated. The distribution of the polar angles of the fibers in the four generated 3D GDLs is shown in Figure 1. The distribution of the carbon fiber pole angles in the reconstructed GDL matched well with that predicted by Equation (13), indicating that the reconstructed GDLs are able to exhibit fiber anisotropy.

In the region under the rib, the GDL is deformed as a result of compression. With an increasing compression ratio, the pore space of the GDL is compressed, and progressively more deformation of the GDL is significant in the area under the rib. The ratio of the reduced thickness to the initial thickness is defined as the compression ratio (CR). By reducing the pore space under the ribs, local compression of the GDL is achieved. The GDL deformation under the GC can be neglected according to the experimental observations and simulations of Jeon et al. [35] on the compressed GDL cross section. See our previous work for details of the compression methodology [47].

**Figure 2:**
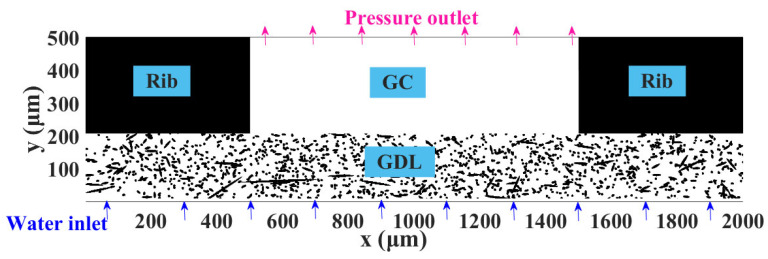
Computational domain including ribs, GC, and GDL; *β* = 1.

The computational domain adopted in the simulation work is the 2D porous GDL with cross-sections selected from the 3D GDL of 2000 × 2000 × 197 μm^3^. The complete computational domain shown in Figure 2 contains the GDL, ribs, and gas channel (GC), with a computational domain size of 2000 × 500 lu^2^; here, 1 lu represents 1 µm [35,36,37]. With an average porosity of 0.78, four GDL structures with different anisotropies are generated. The microstructure of the GDL is presented in Figure 2, with *β* = 1, and the microstructure of the GDL reveals that some of the fibers are oriented at an angle to the through-plane direction, which shows that the adopted two-dimensional computational domain is also capable of showing the structural characteristics of the different anisotropic GDLs and is justified for the study of this paper. The contact angle of the carbon fiber is set to 120° [37,38], corresponding to *w* = 0.013, while the ribs are set to be slightly hydrophobic with a contact angle of 110° [35], corresponding to *w* = 0.010.

All programs were established in MATLAB 2022a (The MathWorks, Natick, MA, USA) and run on a server equipped with two 24-core Intel Xeon Gold 6248R 3.0–4.0GHz CPUs and 128 GB of RAM. No-slip boundary conditions were implemented by applying a half-way bounce-back boundary to all solid surfaces. A computational domain with period boundaries on the left and right planes was established. Based on Zou and He [48], the bottom boundary of the computational domain was set with an inlet flow velocity of 0.001 lu, corresponding to a Reynolds number of 0.042. A sufficiently small dimensionless number indicates that the flow of liquid water in the GDL can neglect viscous effects and that surface tension dominates the behavior of liquid water. The specific implementation of the inlet boundary condition can be given by the following equation:

(14) $$\rho =(f_{9}+f_{1}+f_{3}+2(f_{4}+f_{7}+f_{8}))/(1-v_{in})$$

(15) $$f_{2}=f_{4}+\frac{2}{3}\rho v_{in}$$

(16) $$f_{5}=f_{7}-\frac{1}{2}(f_{1}-f_{3})+\frac{1}{6}\rho v_{in}$$

(17) $$f_{6}=f_{8}+\frac{1}{2}(f_{1}-f_{3})+\frac{1}{6}\rho v_{in}$$

## 4. Results and Discussion

In this paper, the effects of anisotropy and compression on the transport of liquid water within the GDL were investigated by considering four anisotropy coefficients (*β* = 0.1, 1, 10, and 100), along with five compression ratios (0, 10%, 20%, 30%, and 40%). Compression decreases the local porosity of the GDL in the region below the ribs and reduces the volume of the pore space, which leads to changes in transport parameters such as permeability, tortuosity, and diffusivity in the deformed region of the GDL, but in this paper, we were more concerned with the effect of non-uniform deformation caused by compression on the liquid water flow behavior.

### 4.1. Effect of Fiber Anisotropy on Liquid Water Transport within the Uncompressed GDL

**Figure 3:**
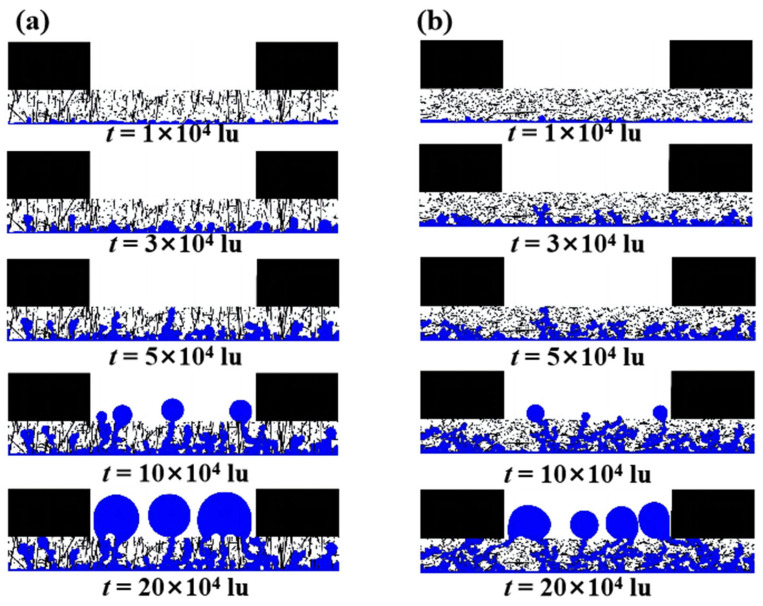

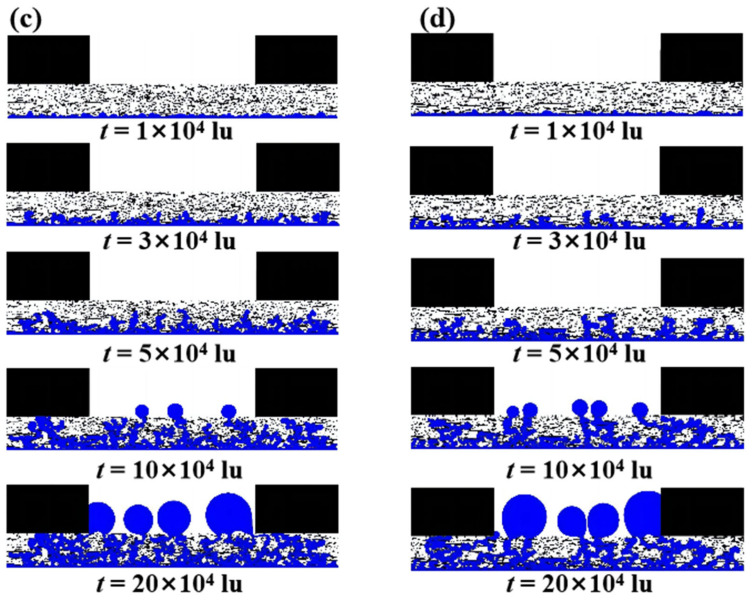
The evolution of the liquid water distribution within the uncompressed GDL with time: (**a**) *β* = 0.1; (**b**) *β* = 1; (**c**) *β* = 10; and (**d**) *β* = 100.

Figure 3 illustrates the evolution of the liquid water distribution within the uncompressed GDL for *β* = 0.1, 1, 10, and 100 with time. Black in Figure 3 represents the solid part (carbon fibers and ribs), and blue represents the liquid water intruding into the GDL from the catalyst layer. At *t* = 1 × 10^4^ lu, which is the initial stage of liquid water transport, liquid water was evenly distributed at the bottom of the GDL and formed a number of bumps. As time passed, the liquid water gradually accumulated within the GDL, and at *t* = 3 × 10^4^ lu, it had flowed into the middle region of the GDLs. According to the Young–Laplace law, the capillary pressure can be calculated as follows:

(18) $$P_{c}=-\frac{\gamma \cos\theta_{c}}{R}$$

where *γ* is the surface tension of the liquid, *θ_c_* is the contact angle of the solid, and *R* is the pore radius. At a certain surface tension and contact angle, capillary pressure and pore radius are inversely related. The smaller the pore, the higher the capillary pressure, so liquid water tends to preferentially fill the large pores, whereas the smaller the anisotropy coefficient, the more fibers tend to be parallel to the through-plane direction (y-direction) and the more localized large pores; thus, more clusters of liquid water were observed within GDLs with *β* = 0.1 and 1 than those with *β* = 10 and 100. At *t* = 5 × 10^4^ lu, the difference in anisotropy led to a more significant difference in the distribution of liquid water. For GDLs with *β* = 0.1 and 1, the end of the liquid water was already flowing towards the top of the GDL, and the liquid water was about to break through the GC, indicating that the angle between the carbon fiber and the in-plane direction (x-direction) accelerated the flow of liquid water within the GDL. The majority of the lateral carbon fibers inside the GDLs with *β* = 10 and 100 had an obstructive effect on the liquid water flow path, which reduced the liquid water flow velocity. At *t* = 10 × 104 lu, the liquid water within all four GDLs broke through to the middle of the GC and formed droplets. As the flow of liquid water continued, some of the clusters of liquid water within the GDL continued to expand, and eventually some droplets formed from liquid water breaking through in the middle region of the GC, while the liquid water breaking through along the rib formed liquid films in contact with the rib, consistent with the phenomenon observed experimentally by Jeon et al. [35].

**Figure 4:**
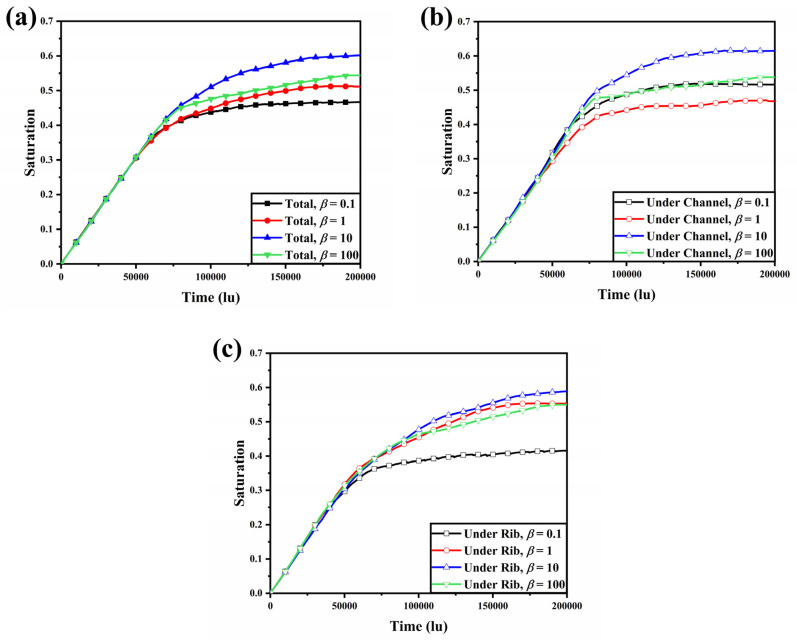
The variation in liquid water saturation over time: (**a**) in the GDL; (**b**) in the GDL under the channel; and (**c**) in the GDL under the rib.

Figure 4 demonstrates the variation in liquid water saturation over time in the GDL, the GDL under the channel, and the GDL under the rib. Liquid water saturation is defined as the proportion of pore space occupied by liquid water. As shown in Figure 4a, liquid water saturation increases linearly until *t* = 5 × 10^4^ lu, due to the fact that the difference in liquid water transport resistance is not significant until the liquid water breaks through to the gas channel. Once the liquid water breaks through to the GC, the pore radius of the liquid water flow pathway will suddenly increase, resulting in a sudden decrease in transport resistance. Thus, after the liquid water pathway is formed, liquid water will preferentially flow out of the pathway to the GC, and the ability to expand in other directions within the GDL will be reduced. After *t* = 5 × 10^4^ lu, the rate of growth of liquid water saturation within the GDL slows down and eventually stabilizes as more flow paths are formed. The liquid water saturation within the two GDLs for *β* = 0.1 and 1 was lower than for *β* = 10 and 100. For the GDLs with *β* = 0.1 and 1, the large proportion of carbon fibers parallel to the through-plane direction or at an inclined angle made it easier to create flow paths when liquid water flowed through. For GDLs with *β* = 10 and 100, however, carbon fibers perpendicular to the through-plane direction dominated, and liquid water was forced to flow laterally along the carbon fibers in search of alternative flow paths. Figure 4b,c show the liquid water saturation within the GDL in the region under the channel and rib, respectively. The liquid water saturation in the GDL under the channel tended to stabilize more easily than that under the ribs, and the liquid water under the ribs could only flow to other directions once it reached the lower end of the rib, until the capillary pressure did not allow it to continue to expand. The significantly lower saturation of liquid water in the under rib region within the GDL for *β* = 0.1 was due to the orientation of the carbon fibers making it easier for the liquid water to flow in the through-plane direction and more difficult to flow in the in-plane direction. The GDL with the fiber orientation parallel to the in-plane direction has been extensively studied in previous research. Steady-state liquid water saturation within the GDL for *β* = 0.1, 1, 10, and 100 was 0.4666, 0.5115, 0.6015, and 0.5440 respectively. Compared to the GDL with *β* = 100, the liquid water saturation in the GDL with *β* = 0.1 decreased by 7.74%, while the GDL with *β* = 10 increased by 10.57%. The effect of GDL anisotropy on liquid water saturation was distinctly demonstrated, where the liquid water within the GDL with *β* = 0.1 broke through to the GC first and with the lowest liquid water saturation.

**Figure 5:**
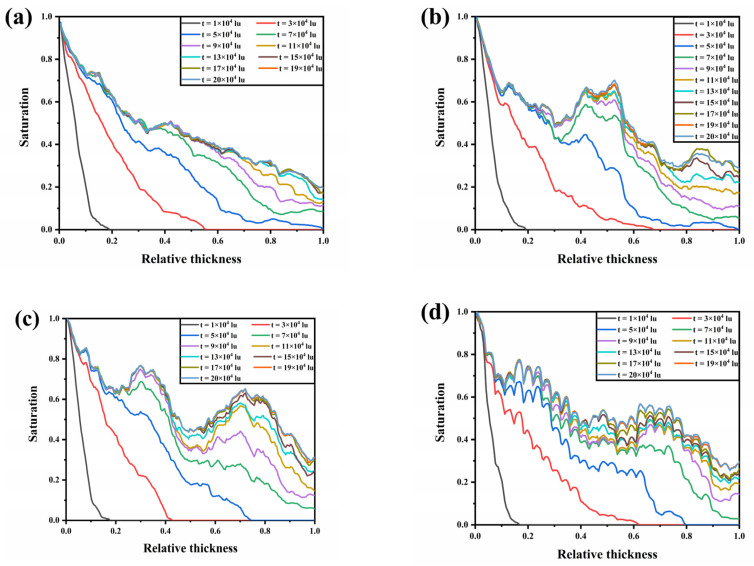
Saturation evolution curves of liquid water in GDLs: (**a**) *β* = 0.1; (**b**) *β* = 1; (**c**) *β* = 10; and (**d**) *β* = 100.

Figure 5 illustrates the variation of liquid water saturation along the thickness direction at different moments. Liquid water saturation increased significantly with time, mainly due to the continued intrusion of liquid water along the through-plane and in-plane directions. The change in the local water saturation curve became insignificant after the liquid water breakthrough due to the fact that the flow resistance of the breakthrough pathway was minimal, and the rate of liquid water expanding new pathways within the GDL slowed down. After that, it was observed that within the four GDLs, the local liquid water saturation curves at moments *t* = 19 × 10^4^ lu and *t* = 20 × 10^4^ lu largely overlapped, indicating that the liquid water flow had reached a steady state. As seen in Figure 5b–d, a peak in local liquid water saturation was observed in the middle of the GDL. This was due to the initial formation of multiple pathways after the liquid water flowed from the catalytic layer into the GDL, and the fibers in the in-plane direction forced the liquid water to flow in the in-plane direction and form water clusters, resulting in an increase in local liquid water saturation. Compared to the GDL with *β* = 1, the GDLs with *β* = 10 and *β* = 100 had more fibers in the in-plane direction, making it more difficult for liquid water to flow in the through-plane direction to form a flow path, and therefore the liquid water aggregation effect was stronger. On the other hand, comparing the GDLs with *β* = 10 and *β* = 100, the near-layered distribution of fibers within the GDL with *β* = 100 and the smaller pores between the fiber layers made it more difficult for liquid water to flow laterally and accumulate within it than in the GDL with *β* = 10. This explains why the GDL with *β* = 10 had the highest liquid water saturation.

### 4.2. Effect of Fiber Anisotropy and Compression on Liquid Water Transport within the GDL

The mechanical stresses introduced during the assembly and integration of the fuel cell system resulted in the compression of the GDL under the ribs. The local porosity of the GDL under the ribs decreased significantly with increasing compression ratio, and changes in microstructure were also evident. However, the effect of compression on the behavior of liquid water transport within anisotropic GDLs needed to be analyzed further.

**Figure 6:**
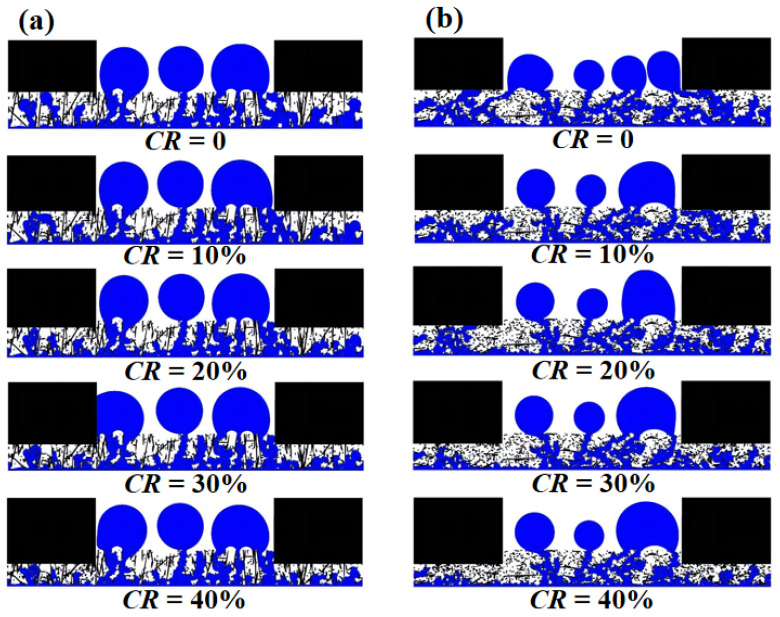

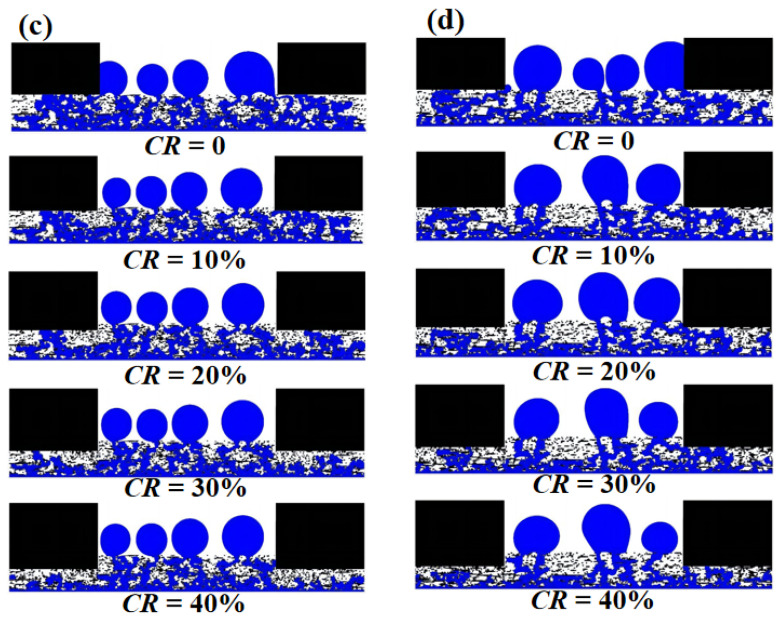
Steady-state liquid water distribution at four different anisotropies of the GDLs at CR = 0, 10%, 20%, 30%, and 40%: (**a**) *β* = 0.1; (**b**) *β* = 1; (**c**) *β* = 10; and (**d**) *β* = 100.

Figure 6 shows the distribution of liquid water at steady state for GDLs with *β* = 0.1, 1, 10, and 100 at compression ratios of 0, 10%, 20% 30%, and 40%. For the uncompressed GDLs, liquid water within the GDLs with *β* = 1, 10, and 100 breakthrough to the GC all presented a liquid film covering the GDL. The significant effect of fiber orientation on liquid water transport was revealed by the fact that liquid water within the sub-rib GDL will flow laterally when contacted by carbon fibers oriented parallel to the in-plane direction and eventually form a pathway across the sub-rib region and the sub-GC region, such that a pathway breaks through to the GC where a liquid film can easily form. It was reported by Jeon et al. [35] that the liquid film is difficult to remove during fuel cell operation and can cover the pathway for reactive gases diffusion from GC to GDL, which is a negative effect on the operation of the fuel cell system. It can be seen that even with 10% compression, the liquid film in the GC in Figure 6b–d disappeared because the interface between the GDL under the GC and the GDL under the rib became more complex due to compression, and the lateral flow of liquid water under the rib was inhibited. As the compression ratio increased, the deformation of the microstructure of the GDL under the rib became more drastic, and the pore size became smaller. Thus, as the compression ratio increased to 20%, 30%, and 40%, the pore size was reduced to the point where it was difficult to support liquid water transport, and the content of liquid water under the rib gradually decreased, which was observed within all four GDLs. Conversely, compression had little effect on the liquid water within the gas diffusion layer under the GC, either on the main circulation path of the liquid water or on the number of droplets within the GC.

**Figure 7:**
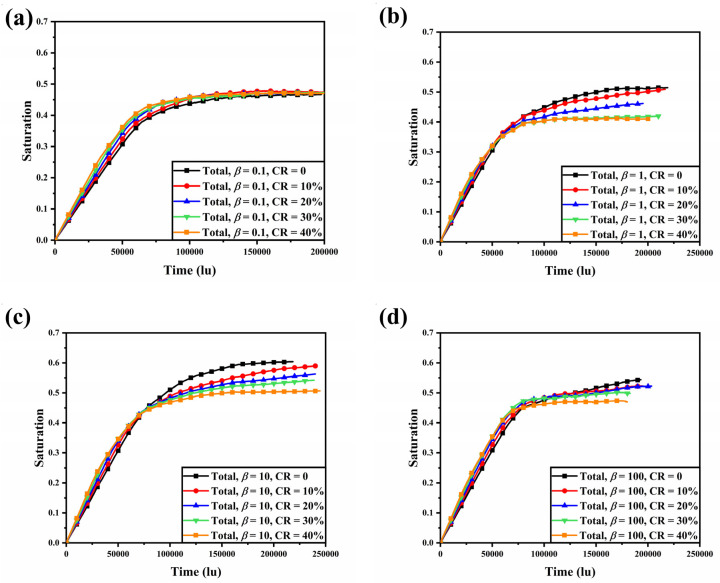
Variation of liquid water saturation with time for four different anisotropies of the GDLs at CR = 0, 10%, 20%, 30%, and 40%: (**a**) *β* = 0.1; (**b**) *β* = 1; (**c**) *β* = 10; and (**d**) *β* = 100.

Variation of liquid water saturation with time for four different anisotropies of the GDLs at CR = 0, 10%, 20%, 30%, and 40% is shown in Figure 7. Similar to the uncompressed condition, the liquid water saturation within the compressed GDL increased nearly linearly until the liquid water broke through. For GDLs with different anisotropy, there was a common trend in liquid water saturation when being compressed: the higher the compression ratio, the faster the initial liquid water accumulation. With the bottom of the GDL set as a constant velocity boundary, the liquid water flux was constant, so water saturation increased at a faster rate when the GDL was compressed, resulting in less pore space. For the GDLs with *β* = 0.1, the steady liquid water saturation was approximately the same for different compression ratios, as the reduction in liquid water content under the ribs was approaching the reduction in pore space. For GDLs with *β* = 1, 10, and 100, compression led to a more significant change in liquid water saturation, which decreased with increasing compression ratio. However, GDLs with different anisotropies had different degrees of influence on liquid water saturation when compressed, which was determined by the morphological characteristics when compressed. At 40% compression of the GDL, the liquid water saturation within the GDL with *β* = 0.1, 1, 10, and 100 was reduced by −0.7%, 20.06%, 16.23%, and 12.26%, respectively, compared to the uncompressed samples. However, at a compression ratio of 40%, the water saturation within the GDL at *β* = 1 was as low as 0.4098. In general, both compression and anisotropy should be considered in order to improve water management in GDLs.

## 5. Conclusions

In this paper, the two-dimensional multiphase LB model is employed to investigate the effects of anisotropy and compression on the transport of liquid water within the gas diffusion layer. The stochastic reconstruction algorithm is adopted to generate GDLs with anisotropy coefficients of *β* = 0.1, 1, 10, and 100, and compression ratios of 0, 10%, 20%, 30%, and 40% are considered. The main conclusions are as follows:

(1) For uncompressed GDLs with different anisotropy, GDLs with *β* = 0.1 and 1 decrease the liquid water saturation compared to GDLs with parallel layers. Carbon fibers angled to the in-plane direction facilitate the formation of the main flow path of liquid water.
(2) Compression leads to structural deformation of the GDL under the rib, the lateral flow of liquid water within the GDL under the rib is prevented, and the liquid film in the GC disappears.
(3) Compression has little effect on the water saturation within the GDL at *β* = 0.1. In contrast, liquid water saturation within GDLs with *β* = 1, 10, and 100 decreases with increasing compression ratio.
(4) GDLs with different anisotropy have different sensitivities to compression, with the lowest liquid water saturation being the GDL with *β* = 0.1 when uncompressed, and the GDL with *β* = 1 at a compression ratio of 40%.

The effects of anisotropy and compression on water management are discussed in this paper, and further research needs to consider more comprehensive models that incorporate heat–electricity–gas transport. On the other hand, due to computational resource constraints and the need to consider the rib-GC structure, this paper simplifies the computational domain to two dimensions. When resources progress further, it would make more sense to extend the computational domain to three dimensions, but this is difficult to achieve at present.

## Data Availability

Not applicable.
